# Association of Impaired Vascular Endothelial Function with Increased Cardiovascular Risk in Asymptomatic Adults

**DOI:** 10.1155/2018/3104945

**Published:** 2018-10-02

**Authors:** Qiuan Zhong, Qingjiao Nong, Baoyu Mao, Xue Pan, Liuren Meng

**Affiliations:** Department of Epidemiology, Guangxi Medical University School of Public Health, 22 Shuangyong Road, Nanning 530021, China

## Abstract

Impaired vascular endothelial function has attracted attention as a prognostic indicator of cardiovascular prevention. The association between impaired endothelial function and cardiovascular risk in the asymptomatic population, however, has been poorly explored. We evaluated the association of brachial artery flow-mediated dilation (FMD) with Framingham-estimated 10-year cardiovascular disease (CVD) risk in subjects free of CVD, especially by cardiovascular risk profiles. In total, 680 adults aged 30-74 years were enrolled from Rongan and Rongshui of Liuzhou, Guangxi, China, through a cross-sectional study in 2015. In the full-adjusted model, the odds ratio for the estimated 10-year CVD risk comparing the low FMD (<6%) with the high FMD (≥10%) was 2.81 (95% confidence interval [CI]: 1.21, 6.53;* P* for trend = 0.03). In subgroup analyses, inverse associations between FMD and the estimated 10-year CVD risk were found in participants with specific characteristics. The adjusted odds ratios, comparing the 25th and the 75th percentiles of FMD, were 2.77 (95% CI: 1.54, 5.00) for aged ≥60 years, 1.77 (95% CI: 1.16, 2.70) for female, 1.59 (95% CI: 1.08, 2.35) for nonsmokers, 1.74 (95% CI: 1.02, 2.97) for hypertension, 1.59 (95% CI: 1.04, 2.44) for normal glycaemia, 2.03 (95% CI: 1.19, 3.48) for C-reactive protein ≥10 mg/L, and 1.85 (95% CI: 1.12, 3.06) for eGFR <106 mL/minute per 1.73 m^2^. Therefore, impaired endothelial function is associated with increased CVD risk in asymptomatic adults. This inverse association is more likely to exist in subjects with higher cardiovascular risk.

## 1. Introduction

The endothelium, a unique endocrine organ, plays an essential role in vascular homeostasis by secreting regulatory factors, such as nitric oxide (NO) [1, 2]. Impairment of endothelial function, mechanistically induced by the loss of NO bioavailability, is strongly regarded as a major contributor to the progression of atherosclerosis by* in vitro* or* in vivo* evidence [3–5]. Notably, prospective cohort studies have shown that brachial artery flow-mediated dilation (FMD), an ultrasonic technique widely used in endothelial function assessment, is a predictor of adverse cardiovascular events in populations with no apparent cardiovascular disease (CVD) [6, 7]. Although impaired endothelial function, as assessed by decreased FMD, is widely suggested as a progressive but reversible manifestation of atherogenesis, the prognostic value of FMD is still controversial in the preventive setting of atherosclerotic CVD [1, 2].

Given the current risk-based guidelines for cardiovascular prevention, a core issue in the prognostic value of FMD is to identify susceptible individuals who potentially respond to endothelial-targeted interventions among subjects free of apparent CVD. Theoretically, impaired endothelial function may be associated with adverse cardiovascular events. However, previous studies have presented discrepant findings in healthy middle-aged men [8–10], hypertensive or healthy postmenopausal women [11–13], and healthy elderly subjects [14, 15], suggesting that the association between endothelial function and cardiovascular risk is not yet well established in the asymptomatic population, especially from the standpoint of CVD risk profiles. In fact, it is ubiquitous that risk factors, such as serum uric acid and oxidized low-density lipoprotein (LDL) cholesterol, play a different role in CVD risk under different population profiles [16].

A recent systematic review with prospective studies indicated that lower brachial FMD may increase cardiovascular risk in asymptomatic populations [17]. However, pooled risk estimates have not yet depicted subjects who are more likely to have a higher cardiovascular risk following decreased FMD. Thus, population profiles are still an open issue regarding the cardiovascular risk linked to impaired endothelial function. In this study, we aimed to evaluate the association of endothelial function with cardiovascular risk as estimated by the Framingham general CVD risk score in subjects free of apparent CVD, especially by different CVD risk profiles.

## 2. Methods

### 2.1. Study Population

We recruited 899 urban and rural adults from Rongan and Rongshui of Liuzhou, Guangxi, China, through a cross-sectional study in 2015. All participants underwent an interview, physical examinations, and laboratory examinations in sequence. Venous blood specimens were obtained after fasting for 12 hours and stored at -20°C until analysis. Among participants, we first restricted the sample to 821 participants aged 30-74 years and then excluded 15 participants with missing brachial FMD measurements, 8 participants with self-reported vasodilator use, and 5 participants with self-reported lipid-lowering treatment. We subsequently excluded 14 participants with an event of general CVD, including myocardial infarction, angina, ischaemic stroke, haemorrhagic stroke, transient ischaemic attack, or peripheral artery disease; 10 participants with other heart diseases; and 89 that were missing other covariates of interest. The final sample included 680 participants. The Medical Ethics Committee of Guangxi Medical University approved the study protocols and all participants provided written informed consent.

### 2.2. FMD Measurement

All the participants abstained from using vasoactive medications, smoking, consuming alcohol, caffeine, or high-fat foods the day before the brachial FMD measurement. After resting at least 15 minutes, measurement was performed using an UNEX EF38G high-resolution ultrasound system (UNEX Corporation, Nagoya, Japan) in a quiet room at a comfortable temperature. Details of the FMD measurement have been described elsewhere [18]. The brachial FMD is expressed as the percent increase in maximum diameter after reactive hyperaemia relative to the baseline brachial artery diameter.

### 2.3. Estimation of 10-Year CVD Risk

Information on age, sex, physician diagnosis of diabetes, use of oral hypoglycaemic medication, insulin, and antihypertension medication, and cigarette smoking was collected by a self-reported questionnaire. Serum glucose, serum total cholesterol, and serum high-density lipoprotein (HDL) cholesterol were determined using a Hitachi automatic analyser 7600-120 or 7600-020 (Hitachi, Tokyo, Japan). Blood pressure was measured using Omron HBP-9021 (Omron, Kyoto, Japan). Diabetes was defined as a self-reported physician diagnosis, a self-reported use of insulin or oral hypoglycaemic medication, or a fasting serum glucose ≥7.0 mmol/L. Smoking status was categorized as never, current, or former. Nonsmoker was defined as fewer than 100 cigarettes smoked in their entire life, current smoker was defined as at least 100 cigarettes smoked in their entire life and reported smoking cigarettes at interview, and former smoker was defined as at least 100 cigarettes smoked in their entire life but with smoking cessation [19].

The estimated 10-year CVD risk for participants aged 30-74 years was calculated using the sex-specific Framingham risk score, which included the covariates of age, total cholesterol and HDL cholesterol concentrations, treated or untreated systolic blood pressure levels, current smoking status (yes, no), and diabetes status (yes, no). The general formula of the equation is as follows:

(1) $$\begin{array}{c} Risk=1-{S_{10}}^{\exp\left(\sum_{i=1}^{n}\beta_{i}{Χ}_{i}-\sum_{i=1}^{n}\beta_{i}{\overset{-}{Χ}}_{i}\right)} \end{array}$$

In the formula, S_10_ is the 10-year baseline survival rate; *β*_i_ is the estimated regression coefficient of the corresponding risk factor; X_i_ is the value of the risk factor, which is 0 or 1 for binary variables and natural log-transformed value for continuous variables; ${\overset{-}{Χ}}_{i}$ is the corresponding mean; and n denotes the number of risk factors. Details of the algorithms for the sex-specific equations were provided elsewhere [20].

### 2.4. Other Variables

Information on education, ethnicity, vasodilator use, lipid-lowering treatment, and physician diagnosis of hypertension was collected by a self-reported questionnaire. Weight and height were measured during the physical examination. Body mass index (BMI) was calculated by dividing weight in kilograms by height in metres squared. Heart rate and baseline brachial artery diameter were monitored using the UNEX EF38G (UNEX Corporation, Nagoya, Japan) during the FMD measurement. Serum triglycerides, serum LDL cholesterol, serum C-reactive protein, and serum creatinine were measured using Hitachi 7600-120 or 7600-020 (Hitachi, Tokyo, Japan). Hypertension was defined as a self-reported physician diagnosis, use of antihypertensive medication, a systolic blood pressure ≥140 mmHg, or a diastolic blood pressure ≥90 mmHg. Dysglycaemia was defined as diabetes or without diabetes but a fasting serum glucose ≥6.1 mmol/L. Elevated lipid levels were defined as ≥200 mg/dL for total cholesterol, ≥150 mg/dL for triglycerides, and ≥130 mg/dL for LDL cholesterol. Low HDL cholesterol was defined as <40 mg/dL. Dyslipidaemia was defined as at least one condition of elevated lipid levels of total cholesterol, triglycerides, or LDL cholesterol or low HDL cholesterol [21, 22]. Serum creatinine was used to calculate an estimated glomerular filtration rate (eGFR) following the arithmetic in the Chronic Kidney Disease Epidemiology Collaboration equation [23].

### 2.5. Statistical Analysis

The brachial FMD was categorized as high (FMD ≥10%), moderate (FMD ≥6% and <10%), or low (FMD <6%), which represent good, moderate, or dysfunctional status in endothelial function, respectively [24–27]. The estimated 10-year CVD risk was categorized as low (Framingham risk score ≤6%), moderate (Framingham risk score >6% and ≤20%), or high (Framingham risk score >20%) [20].

We first used binary logistic regression models to estimate the odds ratios for cardiovascular risk factors comparing the low and moderate categories of FMD to the high category of FMD. Furthermore, the association of endothelial function with estimated 10-year CVD risk was evaluated overall and from subgroups defined by age (<60 years, ≥60 years), sex (male, female), BMI (<24 kg/m^2^, ≥24 kg/m^2^), smoking (never, ever [current and former]), hypertension (yes, no), dysglycaemia (yes, no), dyslipidaemia (yes, no), C-reactive protein (<10 mg/L, ≥10 mg/L), and eGFR (<106 mL/minute per 1.73 m^2^, ≥106 mL/minute per 1.73 m^2^). Ordered logistic regression models were performed to estimate the odds ratios for estimated 10-year CVD risk comparing the low and moderate categories of FMD to the high category of FMD in overall participants and subgroups. We also estimated the odds ratios of estimated 10-year CVD risk by comparing the 25th and 75th percentiles of log-transformed FMD.* P* values for linear trend were obtained by including the medians for each FMD category as continuous variables in the logistic regression models. Finally, we explored the nonlinear relationship between FMD and estimated 10-year CVD risk using restricted quadratic splines with knots at the 10th, 50th, and 90th percentiles of log-transformed FMD.

For the relationship between FMD and estimated 10-year CVD risk, the logistic regression models were progressively adjusted for potential confounders. Model 1 was initially adjusted for age, sex, ethnicity, and education. Model 2 was further adjusted for BMI, smoking status, total cholesterol, HDL cholesterol, serum glucose, C-reactive protein, eGFR, and hypertension. Model 3 was further adjusted for heart rate and baseline brachial artery diameter. Statistical analyses were performed with STATA version 13.1 (StataCorp LP, College Station, TX, USA), and spline functions were conducted in R version 3.4.2 (R Foundation for Statistical Computing, Vienna, Austria). The two-sided statistical significance level was set at *α* = 0.05.

## 3. Results

Overall, the geometric medians of brachial FMD and estimated 10-year CVD risk were 8.2% and 8.3%, respectively, among the 680 study participants. On average, participants with higher estimated 10-year CVD risk were more likely to be older, men, current smokers, dyslipidaemic, hypertensive, and dysglycaemic; to have higher serum total cholesterol, LDL cholesterol, triglycerides, glucose, C-reactive protein, and baseline brachial artery diameter; and to have lower school education, serum HDL cholesterol, eGFR, and FMD** (Table 1)**.

**Figure 1:**
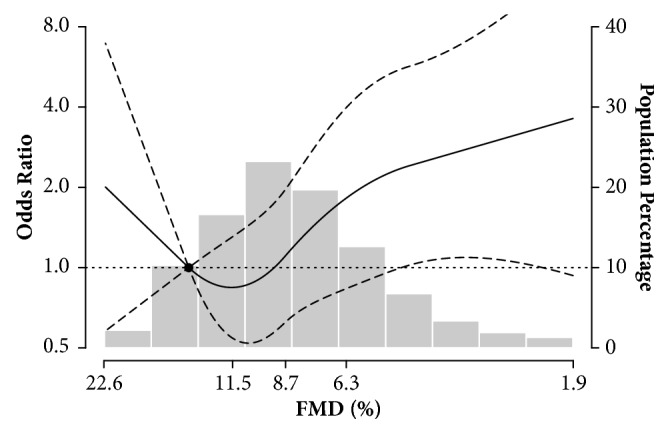
**Odds ratios for estimated 10-year cardiovascular disease (CVD) risk by brachial flow-mediated dilation (FMD). **Odds ratios (solid line) and 95% confidence intervals (curved dashed lines) were based on restricted quadratic splines for log-transformed FMD with knots at the 10th, 50th, and 90th percentiles. The reference (circle) was set at the 90th percentile of FMD distribution. Bars indicate the histogram of FMD distribution in 680 participants. Odds ratios were adjusted for age (years), sex (male, female), ethnicity (Han, Zhuang, other), education (<high school, ≥high school), body mass index (kg/m^2^), smoking status (never, former, current), total cholesterol (mg/dL), high-density lipoprotein cholesterol (mg/dL), serum glucose (mmol/L), C-reactive protein (log mg/L), estimated glomerular filtration rate (mL/minute per 1.73 m^2^), hypertension (yes, no), heart rate (beat per minute), and baseline brachial artery diameter (mm).

There was no significant association between FMD and hypertension, dysglycaemia, or dyslipidaemia after adjustment for age, sex, ethnicity, education, body mass index, C-reaction protein, eGFR, smoking status, heart rate, and baseline brachial diameter (data not shown). For CVD risk, in the model adjusted for age, sex, ethnicity, and education, FMD was not significantly associated with estimated 10-year CVD risk** (Table 2, Model 1;* P* for trend = 0.16)**. Further adjustment for CVD risk factors did not substantially affect this association** (Table 2, Model 2;* P* for trend = 0.32)**. After further adjustment for heart rate and baseline brachial artery diameter, decreased FMD was significantly associated with higher estimated 10-year CVD risk** (Table 2, Model 3;* P* for trend = 0.03)**. The fully adjusted odds ratio for estimated 10-year CVD risk comparing the low FMD (<6%) and the high FMD (≥10%) was 2.81 (95% CI: 1.21, 6.53). The corresponding odds ratio when comparing the 25th and the 75th percentiles of FMD was 1.51 (95% CI: 1.03, 2.20). Additionally, spline regression analysis showed a progressive increase in estimated 10-year CVD risk following decreased FMD from approximately 11.5% (the 75th percentile of FMD distribution)** (Figure 1)**.

For the specified subgroups, significant associations between decreased FMD and higher estimated 10-year CVD risk were found in participants categorized as aged ≥60 years, female, never smokers, normal glycaemia, C-reactive protein ≥10 mg/L, and eGFR <106 mL/minute per 1.73 m^2^ (all* P* for trend <0.05)** (Table 3)**. The odds ratios for estimated 10-year CVD risk, comparing the 25th and the 75th percentiles of FMD, were 2.77 (95% CI: 1.54, 5.00), 1.77 (95% CI: 1.16, 2.70), 1.59 (95% CI: 1.08, 2.35), 1.59 (95% CI: 1.04, 2.44), 2.03 (95% CI: 1.19, 3.48), and 1.85 (95% CI: 1.12, 3.06) for the corresponding characteristics. Additionally, although there were no significant linear trends, estimated 10-year CVD risk was significantly higher when comparing the 25th and the 75th percentiles of FMD in participants with hypertension, and the corresponding odds ratio for estimated 10-year CVD risk was 1.74 (95% CI: 1.02, 2.97).

**Figure 2:**
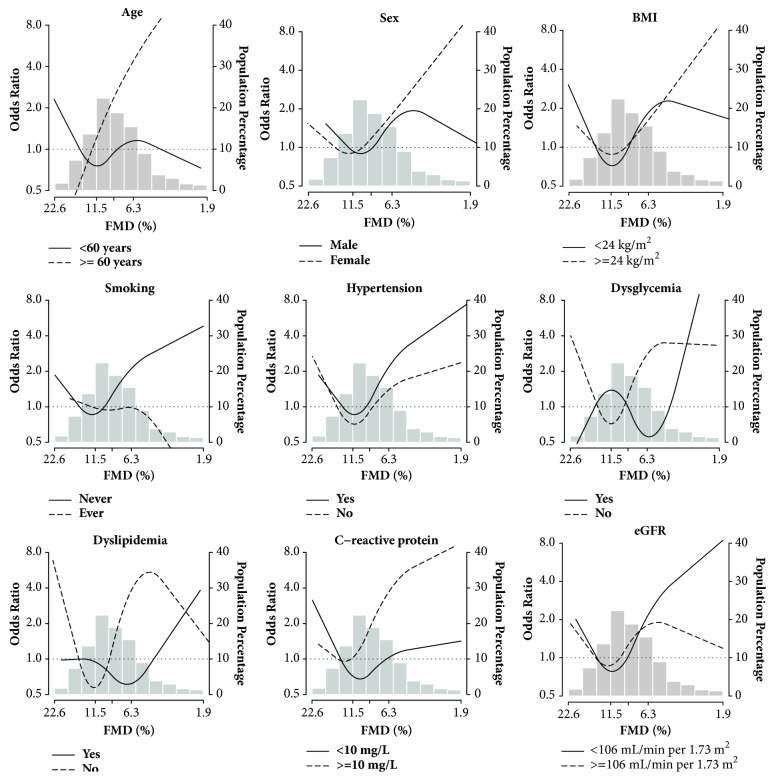
**Odds ratios for estimated 10-year cardiovascular disease (CVD) risk by brachial flow-mediated dilation (FMD) as stratified by participant characteristics.** Odds ratios (solid lines or curved dashed lines) were based on restricted quadratic splines for log-transformed FMD with knots at the 10th, 50th, and 90th percentiles. Bars indicate the histogram of FMD distribution in 680 participants. Odds ratios were adjusted for age (years), sex (male, female), ethnicity (Han, Zhuang, other), education (<high school, ≥high school), body mass index (kg/m^2^), smoking status (never, former, current), total cholesterol (mg/dL), high-density lipoprotein cholesterol (mg/dL), serum glucose (mmol/L), C-reactive protein (log mg/L), estimated glomerular filtration rate (mL/minute per 1.73 m^2^), hypertension (yes, no), heart rate (beat per minute), and baseline brachial artery diameter (mm), except for the factor per se in the corresponding subgroup.

In spline analyses for subgroups, estimated 10-year CVD risk increases following decreased FMD were consistent in participants who were older, female, never smokers, hypertensive, C-reactive protein ≥10 mg/L, and eGFR <106 mL/minute per 1.73 m^2^. It is worth noting that the inverse association between estimated 10-year CVD risk and FMD was present for dysglycaemia and dyslipidaemia, especially within the low FMD (<6%)** (Figure 2)**.

## 4. Discussion

In this study, the reduction of brachial FMD was generally associated with increased cardiovascular risk in subjects free of apparent CVD. Moreover, the inverse associations persisted in subjects with specific profiles, including aged ≥60 years, female, never smokers, hypertensive, C-reactive protein ≥10 mg/L, and eGFR <106 mL/minute per 1.73 m^2^. For subjects with dysglycaemia or dyslipidaemia, the inverse association was more likely to depend on a prerequisite of endothelial dysfunction.

Evidence from experimental and epidemiological studies has confirmed that endothelial dysfunction can be promoted by traditional CVD risk factors (hypertension, dysglycaemia, and dyslipidaemia) [28–32]. Mechanistically, accumulating knowledge recognizes a mutual causality between impaired endothelial function and increased cardiovascular risk. However, the effect of impaired endothelial function on CVD risk factors is less known. For hypertension, a trial in normotensive humans confirmed that intravenous inhibitors of endothelium-derived NO synthase induced large increases in blood pressure [33]. Additionally, a prospective cohort study demonstrated that impaired endothelial function could predict the future development of hypertension in healthy postmenopausal women [12]. In contrast, two community-based prospective cohorts reported that impaired endothelial function did not play a key role in hypertension progression [34, 35]. Consistent with these two studies, the decreased FMD did not significantly relate to hypertension in our study. To our knowledge, no prospective study has suggested an effect of endothelial dysfunction on dysglycaemia or dyslipidaemia. There was also no evidence to support a significant relationship between deteriorated endothelial function and CVD risk factor in our study.

In terms of systematic CVD risk, prospective studies indicate that lower FMD is associated with increased future CVD events in asymptomatic populations [6, 7, 17]. Similarly, cross-sectional studies revealed an inverse relationship between FMD and the 10-year Framingham risk in 5314 Japanese adults [18] and 200 subjects free of coronary heart disease [36]. Across different characteristics, there were inconsistent findings in the association of FMD with cardiovascular risk [8–15]. Notably, the association between FMD and cardiovascular risk was significant in older and postmenopausal women [13, 14] but not in middle-aged men [8, 9]. In general, our study on the association between FMD and cardiovascular risk is in accordance with the previous studies. More importantly, our findings extend study characteristics in the previous studies from sociodemography to cardiovascular risk profiles and indicate a discrepancy in the relationship between endothelial function and cardiovascular risk with different characteristics.

Some underlying mechanisms have been postulated to explain the relationship between impaired endothelial function and increased cardiovascular risk, mainly involving NO bioavailability, oxidative stress, and inflammation [4, 5]. Of these molecular mechanisms, loss of NO bioavailability in the development of endothelial dysfunction is generally accompanied by elevated reactive oxygen species (ROS) and inflammation, which are thought to be the central players in atherosclerosis [37–39]. Meanwhile, previous evidence has shown that endothelial dysfunction can substantially increase cardiovascular risk via pathologic alterations, such as disturbing vascular tone, activating leucocyte migration, promoting blood clotting, and disrupting arterial homeostasis [40–42].

For specific characteristics, ageing or postmenopause mechanistically predisposes individuals to decreased NO bioavailability, increased oxidative stress, inflammation, and atherogenic lipid profiles, contributing to an increased risk of developing CVD [42, 43]. Increasing evidence has also shown that the adverse events of NO bioavailability, oxidative stress, and inflammation similarly exist in diabetes mellitus, hypercholesterolaemia, hypertension, and chronic kidney disease [44–47]. In the present study, 88.4% of women were aged ≥60 years, postmenopausal, hypertensive, high C-reactive protein, low eGFR, dysglycaemic, or dyslipidaemic. Similarly, at least one of the above characteristics was found in 88.6% of nonsmokers or 85.8% of normal glycaemic individuals. Therefore, the subgroup of female, nonsmoking, or normal glycaemia may largely contain the adverse conditions that could alter NO bioavailability, oxidative stress, and inflammation of the vascular endothelium in this study. Regarding the nonsignificant results in ever smokers or dysglycaemia, this may be related to the small size of the study. Briefly, our findings from subgroups suggest that impaired endothelial function is more likely to increase cardiovascular risk in unfavourable cardiovascular profiles that could be promoted by molecular mechanisms, such as loss of NO bioavailability, increased oxidative stress, or inflammation.

There are some limitations in this study. The causal relationship between decreased FMD and increased CVD risk could not be determined due to the cross-sectional design, limiting a reasonable interpretation of decreased FMD as a predictor of increased CVD risk. Moreover, the 10-year CVD risk was estimated using the Framingham general CVD equations that were derived from Western populations, which may not be applicable to Chinese participants, leading to an inaccurate estimation of CVD risk. Although the Framingham general CVD equations have been shown to be relatively appropriate for Chinese populations [48], the current findings still should be interpreted with caution. Finally, FMD is generally susceptible to many factors (dietary, smoking, medication, pathological states, psychophysiological effects, etc.). Most confounding factors had been well controlled for the FMD measurements in this study. However, some potential effects on vasoconstriction derived from inherent factors, such as postmenopause, were difficult to evaluate and overcome, and this may have resulted in a misclassification of FMD.

## 5. Conclusions

Impaired endothelial function was generally associated with increased CVD risk in asymptomatic adults. Most importantly, the inverse association between endothelial function and CVD risk specifically presented in subjects with higher cardiovascular risk status. Growing evidence proposes a prognostic value of vascular endothelial function in both therapy and preventive care [1, 2, 41]. The current findings further suggest that endothelial function may be a favourable prognostic marker for cardiovascular prevention, with an expected benefit in the asymptomatic population at high cardiovascular risk.

## Figures and Tables

**Table 1:** Characteristics of study participants by categories of estimated 10-year CVD risk.

Characteristic	Overall (n=680)	Categories of estimated 10-year CVD risk			*P*_ _^a^
		low risk (n=238)	moderate risk (n=327)	high risk (n=115)	
Age (years)	54.4 (0.4)	45.9 (0.5)	57.3 (0.4)	63.8 (0.7)	<0.001
Male (%)	33.8 (1.8)	12.2 (2.1)	35.5 (2.6)	73.9 (4.1)	<0.001
Ethnicity, Han (%)	19.3 (1.5)	20.2 (2.6)	16.5 (2.0)	25.2 (4.1)	0.11
High school education (%)	11.0 (1.2)	17.2 (2.4)	8.2 (1.5)	6.1 (2.2)	0.001
BMI (kg/m^2^)	23.0 (0.1)	23.0 (0.2)	23.0 (0.2)	23.3 (0.3)	0.55
Current smoking (%)	15.3 (1.4)	3.4 (1.2)	12.2 (1.8)	48.7 (4.7)	<0.001
Total cholesterol (mg/dL)	206.5 (1.9)	190.5 (2.4)	210.2 (2.2)	229.0 (7.3)	<0.001
LDL cholesterol (mg/dL)	113.5 (1.4)	103.7 (1.8)	116.3 (1.8)	126.1 (5.2)	<0.001
HDL cholesterol (mg/dL)	56.1 (0.5)	57.5 (0.9)	56.4 (0.8)	52.3 (1.4)	0.005
Triglycerides (mg/dL)_ _^b^	107.8 (102.8, 113.1)	86.6 (81.0, 92.6)	114.6 (107.2, 122.5)	142.8 (124.7, 163.6)	<0.001
Serum glucose (mmol/L)	5.7 (0.04)	5.4 (0.03)	5.8 (0.05)	6.2 (0.18)	<0.001
C-reactive protein (mg/L)_ _^b^	8.8 (8.4, 9.3)	7.6 (7.2, 8.2)	9.3 (8.7, 10.0)	10.3 (8.9, 11.9)	<0.001
eGFR (mL/minute per 1.73 m^2^)	105.7 (0.6)	115.1 (0.8)	102.7 (0.7)	94.4 (1.6)	<0.001
Dyslipidaemia (%)	62.2 (1.9)	47.5 (3.2)	67.0 (2.6)	79.1 (3.8)	<0.001
Hypertension (%)	42.4 (1.9)	9.7 (1.9)	53.8 (2.8)	77.4 (3.9)	<0.001
Dysglycaemia (%)	21.2 (1.6)	11.3 (2.1)	24.2 (2.4)	33.0 (4.4)	<0.001
Heart rate (beat per minute)	70.0 (0.4)	70.4 (0.6)	69.4 (0.6)	70.9 (1.2)	0.31
Baseline brachial artery diameter (mm)	3.9 (0.02)	3.7 (0.03)	3.9 (0.03)	4.2 (0.05)	<0.001
Brachial FMD (%)_ _^b^	8.2 (7.9, 8.6)	9.2 (8.7, 9.8)	8.1 (7.7, 8.6)	6.7 (6.0, 7.4)	<0.001
Estimated 10-year CVD risk (%)_ _^b^	8.3 (7.8, 9.0)	2.9 (2.7, 3.1)	11.0 (10.6, 11.4)	32.6 (30.5, 34.9)	

Abbreviations: CVD, cardiovascular disease; BMI, body mass index; LDL, low-density lipoprotein; HDL, high-density lipoprotein; eGFR, estimated glomerular filtration rate; FMD, flow-mediated dilation.

^a^One-way analysis of variance, Pearson's chi-square, or Kruskal-Wallis test for differences across categories of estimated 10-year CVD risk.

^b^Geometric means (95% confidence interval). Values in other results are percentages (standard errors) for categorical variables or means (standard errors) for continuous variables unless otherwise indicated.

**Table 2:** Odds ratios (95% confidence interval) for estimated 10-year CVD risk by categories of brachial FMD (n=680).

	FMD (%)			25th versus 75th Percentile	*P* for trend
	≥10 (n=256)	6-10 (n=278)	<6 (n=146)		
Estimated 10-year CVD risk (%)_ _^a^	6.5	8.8	11.4		
Model 1	1 (reference)	1.18 (0.79-1.76)	1.40 (0.87-2.26)	1.07 (0.86-1.33)	0.16
Model 2	1 (reference)	0.96 (0.52-1.77)	1.55 (0.75-3.18)	1.14 (0.83-1.57)	0.32
Model 3	1 (reference)	1.27 (0.66-2.42)	2.81 (1.21-6.53)	1.51 (1.03-2.20)	0.03

Abbreviations: CVD, cardiovascular disease; FMD, flow-mediated dilation.

^a^Geometric means within each category of FMD.

Model 1: adjusted for age (years), sex (male, female), ethnicity (Han, Zhuang, other), and education (<high school, ≥high school).

Model 2: further adjusted for body mass index (kg/m^2^), smoking status (never, former, current), total cholesterol (mg/dL), high-density lipoprotein cholesterol (mg/dL), serum glucose (mmol/L), C-reactive protein (log mg/L), estimated glomerular filtration rate (mL/minute per 1.73 m^2^), and hypertension (yes, no).

Model 3: further adjusted for heart rate (beat per minute) and baseline brachial artery diameter (mm).

**Table 3:** Odds ratios_ _^a^  (95% confidence interval) for estimated 10-year CVD risk by categories of brachial FMD as stratified by characteristics (n=680).

Subgroup	n	FMD (%)			25th versus 75th Percentile	*P* for trend
		≥10 (n=256)	6-10 (n=278)	<6 (n=146)		
Age (years)						
<60	452	1 (reference)	1.03 (0.55-1.92)	1.20 (0.52-2.73)	0.98 (0.66-1.44)	0.71
≥60	228	1 (reference)	2.55 (0.87-7.53)	10.94 (2.58-46.32)	2.77 (1.54-5.00)	0.001
Sex						
Male	230	1 (reference)	0.96 (0.17-5.36)	2.70 (0.29-25.00)	1.15 (0.46-2.89)	0.42
Female	450	1 (reference)	1.30 (0.62-2.74)	4.00 (1.49-10.73)	1.77 (1.16-2.70)	0.02
BMI (kg/m^2^)						
<24	442	1 (reference)	1.18 (0.51-2.73)	2.78 (0.98-7.86)	1.32 (0.81-2.15)	0.08
≥24	238	1 (reference)	1.76 (0.56-5.62)	2.44 (0.46-12.94)	1.84 (0.87-3.91)	0.26
Smoking						
Never	525	1 (reference)	1.31 (0.64-2.66)	3.89 (1.51-10.00)	1.59 (1.08-2.35)	0.01
Ever	155	1 (reference)	0.84 (0.20-3.48)	0.91 (0.18-4.57)	0.70 (0.31-1.59)	0.89
Hypertension						
Yes	288	1 (reference)	1.64 (0.61-4.41)	3.63 (0.97-13.62)	1.74 (1.02-2.97)	0.06
No	392	1 (reference)	1.02 (0.40-2.58)	2.74 (0.82-9.17)	1.27 (0.76-2.14)	0.18
Dysglycaemia						
Yes	144	1 (reference)	0.94 (0.22-3.94)	3.39 (0.52-22.31)	1.85 (0.87-3.95)	0.31
No	536	1 (reference)	1.67 (0.77-3.62)	3.56 (1.33-9.54)	1.59 (1.04-2.44)	0.02
Dyslipidaemia						
Yes	423	1 (reference)	0.63 (0.31-1.26)	1.02 (0.42-2.46)	1.13 (0.71-1.79)	0.70
No	257	1 (reference)	2.34 (0.70-7.90)	4.26 (0.98-18.55)	1.20 (0.68-2.13)	0.05
C-reactive protein (mg/L)						
<10	436	1 (reference)	0.92 (0.38-2.18)	1.37 (0.43-4.34)	1.08 (0.63-1.86)	0.69
≥10	244	1 (reference)	1.80 (0.61-5.30)	7.55 (2.04-27.97)	2.03 (1.19-3.48)	0.005
eGFR (mL/minute per 1.73 m^2^)						
<106	337	1 (reference)	0.88 (0.35-2.19)	4.99 (1.54-16.15)	1.85 (1.12-3.06)	0.03
≥106	343	1 (reference)	3.19 (1.03-9.92)	1.36 (0.31-5.90)	1.19 (0.60-2.37)	0.40

Abbreviations: CVD, cardiovascular disease; FMD, flow-mediated dilation; BMI, body mass index; eGFR, estimated glomerular filtration rate.

^a^Results were adjusted for age (years), sex (male, female), ethnicity (Han, Zhuang, other), education (<high school, ≥high school), body mass index (kg/m^2^), smoking status (never, former, current), total cholesterol (mg/dL), high-density lipoprotein cholesterol (mg/dL), serum glucose (mmol/L), C-reactive protein (log mg/L), estimated glomerular filtration rate (mL/minute per 1.73 m^2^), hypertension (yes, no), heart rate (beat per minute), and baseline brachial artery diameter (mm), except for the factor per se in the corresponding subgroup.

## Data Availability

The data used to support the findings of this study are available from the corresponding author upon request.
